# Correlation between research productivity during and after orthopaedic surgery training^☆^

**DOI:** 10.1016/j.sopen.2024.02.010

**Published:** 2024-02-25

**Authors:** Daniel Acevedo, Henson Destiné, Christopher J. Murdock, Dawn LaPorte, Amiethab A. Aiyer

**Affiliations:** a Nova Southeastern University Dr. Kiran C. Patel College of Allopathic Medicine (NSU MD), United States of America; b University of Miami Leonard M. Miller School of Medicine, United States of America; c Johns Hopkins Medicine Department of Orthopaedic Surgery, United States of America

**Keywords:** Orthopaedic surgery, Residency, Resident education, Research productivity, Research involvement

## Abstract

**Background:**

Research experience is mandatory for all Orthopaedic Surgery residency programs. Although the allocation of required protected time and resources varies from program to program, the underlying importance of research remains consistent with mutual benefit to both residents and the program and faculty. Authorship and publications have become the standard metric used to evaluate academic success. This study aimed to determine if there is a correlation between the research productivity of Orthopaedic Surgery trainees and their subsequent research productivity as attending Orthopaedic Surgeons.

**Methods:**

Using the University of Mississippi Orthopaedic Residency Program Research Productivity Rank List, 30 different Orthopaedic Surgery Residency Programs were analyzed for the names of every graduating surgeon in their 2013 class. PubMed Central was used to screen all 156 physicians and collect all publications produced by them between 2008 and August 2022. Results were separated into two categories: Publications during training and Publications post-training.

**Results:**

As defined above, 156 Surgeons were analyzed for publications during training and post-training. The mean number of publications was 7.02 ± 17.819 post-training vs. 2.47 ± 4.313 during training, *P* < 0.001. The range of publication post-training was 0–124 vs. 0–30 during training. Pearson correlation between the two groups resulted in a value of 0.654, *P* < 0.001.

**Conclusion:**

Higher research productivity while training correlates to higher productivity post-training, but overall Orthopaedic surgeons produce more research after training than during. With the growing importance of research, more mentorship, time, and resources must be dedicated to research to instill and foster greater participation while in training.

## Highlights

• Residents who do more research tend to continue producing as attendings.
• Overall surgeons produce more research after training than during.
• Most residents did not produce published works.

## Introduction

Research experience and participation is a mandatory component of all orthopaedic Surgery residency programs as outlined by the Accreditation Council for Graduate Medical Education (ACGME). Each program is required to provide a minimum of 60 days of protected research time, instruction in research methodology, and sufficient resources. Additionally, residents of accredited programs are mandated to participate in research, publish research, or present research with the number of publications used as a common criterion to evaluate academic productivity [[1], [2], [3]]. Although the allocation of required protected time and resources varies from program to program, the underlying importance of research remains consistent with mutual benefit to both residents and the program and faculty [4]. Resident participation in research has improved clinical care by increasing their comfort in evidence-based medicine and enhancing their analytical and critical thinking skills; in addition, research participation facilitates the acquisition of a mentor, which has been deemed to be a crucial aspect of medical education, professional development, and research success itself [[5], [6], [7], [8]]. Residency programs benefit from the improved program reputation and academic standing that resident research participation creates [6,7]. As a result, authorship and publications have become a standard metric for evaluating admission to medical school, residency, fellowship, promotion in rank, perceived expertise, and even membership into professional societies [[8], [9], [10]].

Lack of standardization allows for differing interpretations of ACGME core requirements across programs, resulting in varied protected research time, requirements, and institutional support [11]. As such, research productivity amongst residents at different programs and even within the same program is widely varied based on the implementation of a program's chosen research structure within ACGME guidelines [8]. A foundational goal of orthopaedic training is to produce graduates able to contribute to orthopaedic knowledge and advance the field, a task accomplished through research or academic-based teaching careers [3,6]. Studies conducted within plastic surgery have found residents who were more active in research were associated with being more academically productive once they are attendings, with increased numbers of publications during training being predictive of a greater total number of publications after training [4]. The association between research productivity during residency and research productivity after orthopaedic surgery training has not been clearly illustrated.

This study aims to determine if there is an association between the number of peer-reviewed articles published by orthopaedic surgery trainees and their subsequent publication rates 6 years into practice. Following the results seen within plastic surgery, we hypothesize that there will be a positive correlation between the research productivity of trainees and practicing surgeons.

## Methods

The University of Mississippi Medical Center (UMMC) Orthopaedic Surgery and Rehabilitation Department published a list titled “Orthopaedic Residency Program Research Productivity Rank List” (Supplemental Table 1) [12]. This list breaks down orthopaedic residency programs by “Total Citations” and “Number of Publications”. It uses those to assign them an impact rank to the residency based on the cumulative research productivity of the residency program. The full details on how this list, and the subsequent database it is based on, was made is published in Jones et al., 2018 [13]. Beginning at the top of the list, 2 authors (D.A, H·D) conducted searches on each program until data was collected on 30 orthopaedic surgery residency programs. Programs were excluded if they did not have their 2013 alumni class published, required special permissions to access their alumni information, or were too new a program to have a 2013 residency class. The class of 2013 was chosen because at the time of writing it was the most recent class which would have an equal number of years spent within orthopaedic training as years spent post-training; the intention being that the study results would be more reflective of recent trends in publication. Ultimately, 51 programs were screened (∼1/3 of available programs) before reaching the included 30 orthopaedic surgery residency programs; 21 programs were excluded according to our exclusion criteria (Table 1). The included programs were analyzed for the names of every graduating surgeon in their 2013 class. 157 surgeons were collected, 1 excluded due to not completing training, and 156 were included for final analysis. Their names were recorded directly from their respective program's website.

The U.S. National Institute of Health's National Library of Medicine (NIH/NLM) database via PubMed Central was used to screen all 156 physicians and collect all publications produced by them between 2008 (the year they started residency) and August 2022. Physician names were searched in the following format: “First Name, Middle initial, Last Name, Orthopaedic.” University and program affiliation tags were primarily used to potentially screen out articles by other authors who shared the same name. Results were separated into two categories: Publications during training and Publications post-training. Training was defined as any time spent in residency and fellowship. Orthopaedic surgery residencies are 60 months (5 years), with some programs offering an optional 12 months (1 year) for research. Residency and Fellowship graduation data was collected for each physician. The categories were subsequently defined as Publications during training = 2008–2014, the additional year was added to account for the fellowship graduation date of 154 of the 156 physicians, and Publications post-training =2015–2022. An exception was made for 2 of the 156 physicians who completed two separate fellowships. For them, the categories were defined as Publications during training = 2008–2015 and Publications post-training = 2016–2022.

**Table 1:** The top 30 programs included in our analysis and their corresponding impact rank according to The University of Mississippi Medical Center's “Orthopaedic Residency Program Research Productivity Rank List”.

Program #	UMMC's impact rank	Program name
1.	1	Massachusetts General Hospital/Brigham and Women's Hospital/Harvard Medical School
2.	6	Rush University Medical Center
3.	7	University of California (San Francisco)
4.	9	University of Washington
5.	10	Stanford University
6.	11	Boston University Medical Center
7.	12	University of Minnesota
8.	16	Duke University Hospital
9.	17	University of Pennsylvania
10.	19	Johns Hopkins
11.	20	University of Utah
12.	21	University of Michigan
13.	23	University of Virginia
14.	24	University of California (San Diego)
15.	28	Ohio State University Hospital
16.	32	Vanderbilt University Medical Center
17.	34	University of South Florida Morsani
18.	36	Carolinas Medical Center
19.	38	Yale-New Haven Medical Center
20.	39	Emory University
21.	41	University of Vermont Medical Center
22.	42	University of California (Davis) Health System
23.	43	University of Maryland
24.	45	University of Rochester
25.	46	University of Florida
26.	47	University of Louisville
27.	48	Wake Forest University School of Medicine
28.	49	Naval Medical Center (Portsmouth)
29.	50	Baylor College of Medicine
30.	51	Dartmouth-Hitchcock Medical Center

Statistical significance was set at *P* values <0.05 for all statistical analyses conducted using SPSS (IBM SPSS Statistics version 28). Chi-square analysis, *t*-tests, and ANOVA were used for the appropriate categorical and continuous variables.

## Results

As defined above, 156 Surgeons were analyzed for publications during training and post-training. The mean number of publications was significantly higher post-training than during training (7.02 ± 17.819 post-training vs. 2.47 ± 4.313 during training, *P* < 0.001). There was a greater range of publications post-training as opposed to during training (0–124 post-training vs. 0–30 during training), but both groups shared the same mode (0 publications for both groups with 55 surgeons classified as 0 publications during training and 46 surgeons classified as 0 publications post-training). Pearson correlation between the two groups (during training and post-training) resulted in a value of 0.654 [95 % confidence interval; 0.554–0.736; lower – higher] (*P* < 0.001). Of note, only 16 surgeons reported 0 publications during the entire study period of 2008 to 2022 and thus were marked as having produced 0 publications during both training and post-training.

**Fig. 1:**
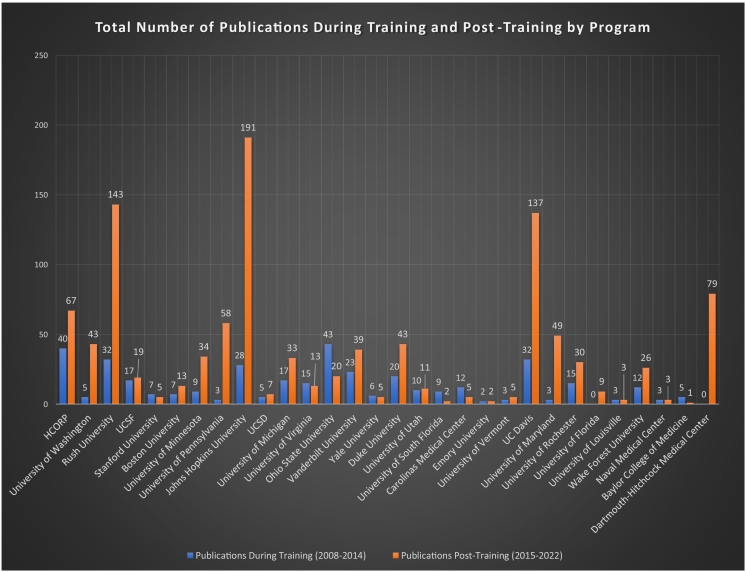
Total number of publications during training and post-training by program.

Further sub-analysis was conducted to determine if a statistical difference existed due to the residency program attended. The 156 surgeons were grouped into their respective residency programs and analyzed for differences between the different programs through computed ANOVA tests. Analysis was conducted on the groups both during training and post-training with identical group composition (grouping variable = residency attended) for both. There was no significant difference between the research productivity of the different residency programs' 2013 class (as evaluated by means of the program based on the productivity of the 2013 class during their entire training length) nor the subsequent research productivity of the alumni produced by those programs(the individuals were left stratified within their original group based on residency and then evaluated within the post-training period) (F value of 1.043, *P* = 0.419 during residency, and F value of 1.251, *P* = 0.199 post-residency). Total publications by residency program can be seen in Fig. 1. Further tests of Homogeneity of Variances between the groups revealed that assumptions were violated, and additional Kruskal-Wallis tests were conducted for non-parametric data. The Kruskal- Wallis test also utilized the Residency Program attended as the grouping variable. Similarly, it showed that no significant differences existed between the research productivity of the different residency programs evaluated and their alumni's subsequent productivity (*P* = 0.066 during training and *P* = 0.198 post-training).

## Discussion

Overall, despite different research requirements across programs, there is no significant difference between the research productivity of different residency programs nor the ensuing productivity of their comprehensive alumni. Despite similar modes, surgeons tend to publish more after training than they do during training, with a significantly strong correlation between how much they publish during training and how much they publish post-training.

Examination of different program websites and the published curriculum outline confirms that the research structure is not uniform between different programs. Dedicated research time can vary from the minimum required 60 days to much longer times based on the institution. This is true of the 30 different programs analyzed in this study. This difference in protected time however did not translate to a significant difference in the number of publications seen between the different residency programs in our study as evidenced by our Kruskal-Wallis results. While the sample size for each program in our study was small given that it represented only one cohort of residents, Osborn et al. found analogous results showing that despite the length of protected research time, publication amount and quality of publications did not differ between different orthopaedic residency programs. Comparatively, Osborn et al. reported that what did increase, was the number of publications produced by the outliers compared to the majority, thus suggesting that increased research time allows highly motivated people to complete and produce more projects. [11] During training, this study produced a range of 0–30 publications, with 55 surgeons making up the mode of 0 publications. Our maximum value of 30 was achieved by 1 surgeon, with the second highest productivity in his program being 1. Our next four highest values within training were 26, 20, 18, and 15 but all were at a different program amongst the 30. Within the program those values belonged to, the resident with the next highest levels of productivity had values of 5, 8, 5, and 7 respectively. Furthermore, if you remove the 5 highest reported numbers of productions as outliers (30, 26, 20, 18, 15, all at a different institution), of the remaining 151 surgeons, 149 of them produced 8 or less publications hence supporting Osborn et al. finding's above.

The mean number of publications post-training was significantly higher than the mean while training. Several different reasons could account for this but when asked, residents have listed lack of time, insufficient research skills, the intensity of clinical duties, financial support, and inadequate mentorship as the major barriers to project completion [4,[14], [15], [16]]. Due to a growing pressure to increase clinical activities, more than half of surgeons report spending <20 % of their efforts on research creating a visible lack of modeling and mentorship within programs for residents [17].

There was a strong correlation between surgeons who produced while training and their propensity to produce and continue to produce after training. The range for research productivity after training is 0–124 with a similar mode of 0. In decreasing order, the 5 highest values were 124, 118, 103, 49, and 47. Of note, surgeons who pursue an academic career following successful completion of training have been found to publish more while training than those who pursue alternative career paths [2,15]. Four of those values listed previously belong to surgeons working in academic settings with the remainder classified as “privademics”. Increased levels of research productivity are common in academic settings; higher levels lead to higher clinical volumes, increased personal and institutional reputations, and higher levels of perceived expertise; consequently, publications and impact are elevated as the driving force behind advancement in academic medicine [4,18]. The academic setting also provides advantages such as resident assistance on research projects or the presence of research fellows to carry the project forward while attendings focus on clinical obligations. Clark et al. published a study on the impact of a research fellow and found that the average number of publications gained from completion of a research year was 10.8 [19]. Every publication that a research fellow successfully brings to print is overseen by an attending surgeon and many of the programs outlined in that study accept >1 fellow, thus exponentially increasing productivity. Surgeons who have the benefit of a research fellow(s) or even the combined assistance of a resident, clinical fellow, and research fellow would be more primed to report higher productivity. Several of the programs within this study were mentioned in the article written by Clark et al. as having dedicated research fellowships with others being found on Orthogate; a few attendings evaluated in this study even have specific research fellowship programs that they oversee as the principal investigator (PI). Comparisons between the surgeons listed as having research fellowships and their eventual post-training productivity suggest that the increased resources (such as research fellows) play a role in their post-training productivity.

The results of this study have brought to light the importance and influence of longitudinal involvement in research, a “research pipeline”. Multiple studies have positively correlated research before residency leading to an inclination to do research during residency. Residents who conducted research during medical school and have advanced degrees that integrated research or prior research experiences such as dedicated research years are all more likely to publish and be involved with research during training [8,[20], [21], [22], [23]]. This study further adds to these findings with significant correlations and findings showing a continued trend between research while training and research involvement post-training. While this creates the illusion of a pipeline starting with research before training, through training, and post-training, a separate study with one cohort is needed to confirm if early exposure and involvement during medical school can have lasting effects extending into future practice. Inadequate research training, as well as lack of interest in research during residency, has been shown to contribute to limited productivity as practicing orthopaedic clinicians, therefore focus should be placed on establishing more research personnel to be available as mentors and more formal education in research to increase resident competency and participation in research and research output [7,11].

This study is not without limitations. First, only PubMed publications were used and recorded to determine publication and scholarly output for all time points within this study, potentially missing data from other sources such as book chapters and non-peer-reviewed publications. Additionally, given how the search phrase in PubMed was formatted, publications from these surgeons that were not orthopaedic-related may not have been recorded. As a result, we may be underestimating the number of publications for surgeons in this study. Second, research projects are continuous works that may be published at any time point regardless of when the project began. As a result, research started while in medical school may not be published until residency training or research worked on while training may not be published until post-training, especially if research years were taken; similarly, fellowship-completed research projects would influence post-training productivity. This would potentially affect the numbers seen above for both categories, to hedge against a potential bias towards the post-training group we included all articles published during the fellowship graduation year as part of the “in training” group, allotting up to an additional 6 months for projects that were already submitted to reach publication. Next, this study included 156 surgeons over 30 programs and represents a small sample size in comparison to the general population of orthopaedic surgeons. This small sample size is most concerning for type B error in our comparison of the 30 respective residency programs' research productivity. Due to the inherent nature of orthopaeidc surgery programs being smaller in class size than non-surgical specialties, future studies would need to span multiple classes in order to ascertain if there truly is no difference amongst programs. Despite these limitations, we believe our study provides convincing evidence and represents honest work.

## Conclusion

Higher research productivity while training is strongly associated with higher productivity rates post-training, but overall orthopaedic surgeons produce more research after training than during. If basic science and clinical research is deemed to be a critical component of resident and fellowship education then our specialty should consider dedicating more time and money to developing research scholarly activity.

The following is the supplementary data related to this article.

Supplemental Table 1 Copy of The University of Mississippi Medical Center's “Orthopaedic Residency Program Research Productivity Rank List”
Source: Jones et al., “Objective Methodology to Assess Meaningful Research Productivity by Orthopaedic Residency Departments: Validation Against Widely Distributed Ranking Metrics and Published Surrogates”, 2018
Used with permission granted by Elsevier. Supplemental Table 1

## Funding

No funding or support was received for any aspect of this work.

## Ethical approval statement

This was an IRB-exempt study.

## CRediT authorship contribution statement

**Daniel Acevedo:** Conceptualization, Data curation, Writing – original draft. **Henson Destiné:** Data curation, Formal analysis, Writing – original draft. **Christopher J. Murdock:** Conceptualization, Methodology, Writing – review & editing. **Dawn LaPorte:** Methodology, Supervision, Writing – review & editing. **Amiethab A. Aiyer:** Conceptualization, Methodology, Supervision, Writing – review & editing.

## Declaration of competing interest

The authors declare that they have no known competing financial interests or personal relationships that could have appeared to influence the work reported in this paper.
