# Supplementary material for: Correlation between research productivity during and after orthopaedic surgery training
Source: Surg Open Sci. 2024 Feb 25;18:98–102. doi: 10.1016/j.sopen.2024.02.010 (PMC10910153; doi:10.1016/j.sopen.2024.02.010)
Supplement: Supplemental Table 1 — Copy of The University of Mississippi Medical Center's “Orthopaedic Residency Program Research Productivity Rank List” Source: Jones et al., “Objective Methodology to Assess Meaningful Research Productivity by Orthopaedic Residency Departments: Validation Against Widely Distributed Ranking Metrics and Published Surrogates”, 2018 Used with permission granted by Elsevier. [file mmc1.docx]

| Impact  Rank | Program Name | Total Citations | Number of  Publications |
| --- | --- | --- | --- |
| 1 | Massachusetts General Hospital/Brigham and Women's Hospital/Harvard Medical School | 40,122 | 2,579 |
| 2 | Hospital for Special Surgery/Cornell Medical Center | 35,556 | 2,206 |
| 3 | Washington University/B-JH/SLCH Consortium | 26,005 | 1,458 |
| 4 | Mayo Clinic College of Medicine (Rochester) | 22,805 | 1,586 |
| 5 | UPMC Medical Education | 21,570 | 1,080 |
| 6 | Rush University Medical Center | 20,049 | 1,163 |
| 7 | University of California (San Francisco) | 17,686 | 986 |
| 8 | Thomas Jefferson University | 16,687 | 1,155 |
| 9 | University of Washington | 16,566 | 893 |
| 10 | Stanford University | 15,463 | 879 |
| 11 | Boston University Medical Center | 12,873 | 561 |
| 12 | University of Minnesota | 12,748 | 656 |
| 13 | New York University School of Medicine/Hospital for Joint Diseases | 12,470 | 870 |
| 14 | UCLA Medical Center | 12,066 | 637 |
| 15 | Cleveland Clinic Foundation | 11,925 | 771 |
| 16 | Duke University Hospital | 11,860 | 753 |
| 17 | University of Pennsylvania | 11,350 | 982 |
| 18 | University of Iowa Hospitals and Clinics | 10,626 | 654 |
| 19 | Johns Hopkins | 10,335 | 823 |
| 20 | University of Utah | 10,156 | 726 |
| 21 | University of Michigan | 10,076 | 813 |
| 22 | University of North Carolina Hospitals | 9,076 | 518 |
| 23 | University of Virginia | 9,039 | 581 |
| 24 | University of California (San Diego) | 8,852 | 540 |
| 25 | University of Wisconsin | 8,438 | 461 |
| 26 | New York Presbyterian Hospital (Columbia Campus) | 8,395 | 610 |
| 27 | University of Southern California/LAC+USC Medical Center | 8,108 | 556 |
| 28 | Ohio State University Hospital | 7,984 | 591 |
| 29 | University of Cincinnati Medical Center/College of Medicine | 7,136 | 330 |
| 30 | University of Colorado | 6,969 | 524 |
| 31 | McGaw Medical Center of Northwestern University | 6,827 | 526 |
| 32 | Vanderbilt University Medical Center | 6,644 | 473 |
| 33 | Case Western Reserve University/University Hospitals Case Medical Center | 6,478 | 480 |
| 34 | University of South Florida Morsani | 6,269 | 380 |
| 35 | Indiana University School of Medicine | 5,985 | 315 |
| 36 | Carolinas Medical Center | 5,727 | 342 |
| 37 | University of California (Irvine) | 5,701 | 268 |
| 38 | Yale-New Haven Medical Center | 5,653 | 399 |
| 39 | Emory University | 5,515 | 325 |
| 40 | National Capital Consortium | 5,335 | 334 |
| 41 | University of Vermont Medical Center | 5,323 | 226 |
| 42 | University of California (Davis) Health System | 5,271 | 396 |
| 43 | University of Maryland | 5,230 | 407 |
| 44 | University of Connecticut | 5,201 | 298 |
| 45 | University of Rochester | 5,183 | 432 |
| 46 | University of Florida | 5,044 | 297 |
| 47 | University of Louisville | 4,936 | 288 |
| 48 | Wake Forest University School of Medicine | 4,372 | 271 |
| 49 | Naval Medical Center (Portsmouth) | 4,250 | 289 |
| 50 | Baylor College of Medicine | 4,148 | 281 |
| 51 | Dartmouth-Hitchcock Medical Center | 4,060 | 212 |
| 52 | San Antonio Uniformed Services Health Education Consortium (SAUSHEC) | 3,902 | 299 |
| 53 | Tufts Medical Center | 3,734 | 225 |
| 54 | Oregon Health & Science University | 3,668 | 252 |
| 55 | University of Kentucky College of Medicine | 3,637 | 301 |
| 56 | University of Illinois College of Medicine at Chicago | 3,619 | 288 |
| 57 | Rutgers New Jersey Medical School | 3,483 | 232 |
| 58 | Brown University | 3,403 | 323 |
| 59 | University of Alabama Medical Center | 3,264 | 239 |
| 60 | Henry Ford Hospital/Wayne State University | 3,208 | 193 |
| 61 | Baylor University Medical Center | 3,156 | 187 |
| 62 | University of Massachusetts | 2,988 | 168 |
| 63 | University of Hawaii | 2,967 | 68 |
| 64 | Loyola University | 2,932 | 299 |
| 65 | University of Texas Southwestern Medical School | 2,929 | 242 |
| 66 | Temple University Hospital | 2,882 | 253 |
| 67 | University of Texas Health Science Center School of Medicine at San Antonio | 2,867 | 143 |
| 68 | Virginia Commonwealth University Health System | 2,769 | 183 |
| 69 | Medical College of Wisconsin Affiliated Hospitals | 2,703 | 191 |
| 70 | University of Chicago | 2,534 | 171 |
| 71 | Greenville Health System/University of South Carolina | 2,402 | 214 |
| 72 | Grand Rapids Medical Education Partners/Michigan State University | 2,390 | 180 |
| 73 | Union Memorial Hospital | 2,275 | 190 |
| 74 | University of Missouri-Columbia | 2,264 | 158 |
| 75 | Jackson Memorial Hospital/Jackson Health System | 2,192 | 215 |
| 76 | William Beaumont Hospital | 2,191 | 163 |
| 77 | Penn State Milton S Hershey Medical Center | 2,113 | 158 |
| 78 | University of Nebraska Medical Center College of Medicine | 2,067 | 149 |
| 79 | University of Kansas School of Medicine | 1,987 | 144 |
| 80 | Cedars-Sinai Medical Center | 1,983 | 99 |
| 81 | Montefiore Medical Center/Albert Einstein College of Medicine | 1,929 | 178 |
| 82 | University of Tennessee | 1,784 | 136 |
| 83 | University of Toledo | 1,753 | 143 |
| 84 | Icahn School of Medicine at Mount Sinai/St Luke's- Roosevelt Hospital Center | 1,731 | 104 |
| 85 | William Beaumont Army Medical Center/Texas Tech University (El Paso) | 1,632 | 133 |
| 86 | St Louis University School of Medicine | 1,600 | 154 |
| 87 | Texas Tech University (Lubbock) | 1,510 | 126 |
| 88 | Allegheny Health Network Medical Education Consortium (AGH) | 1,500 | 108 |
| 89 | University at Buffalo | 1,419 | 61 |
| 90 | University of New Mexico | 1,392 | 88 |
| 91 | Icahn School of Medicine at Mount Sinai | 1,370 | 133 |
| 92 | Loma Linda University | 1,343 | 98 |
| 93 | West Virginia University | 1,272 | 114 |
| 94 | Drexel University College of Medicine/Hahnemann University Hospital | 1,254 | 99 |
| 95 | University of Mississippi Medical Center | 1,223 | 109 |
| 96 | Hofstra North Shore-LIJ School of Medicine at Lenox Hill Hospital | 1,221 | 70 |
| 97 | Medical University of South Carolina | 1,217 | 118 |
| 98 | University of Texas Medical Branch Hospitals | 1,212 | 114 |
| 99 | George Washington University | 1,161 | 102 |
| 100 | Geisinger Health System | 1,131 | 108 |
| 101 | Mount Carmel Health System | 1,095 | 87 |
| 102 | SUNY Upstate Medical University | 1,084 | 104 |
| 103 | University of Arizona | 1,076 | 106 |
| 104 | Georgetown University Hospital | 1,050 | 115 |
| 105 | Stony Brook Medicine/University Hospital | 998 | 85 |
| 106 | University of Oklahoma Health Sciences Center | 983 | 73 |
| 107 | Tulane University | 933 | 74 |
| 108 | Medical College of Georgia | 872 | 47 |
| 109 | Texas A&M College of Medicine-Scott and White | 859 | 69 |
| 110 | Methodist Hospital (Houston) | 816 | 80 |
| 111 | Tripler Army Medical Center | 813 | 57 |
| 112 | Louisiana State University | 766 | 69 |
| 113 | University of Arkansas for Medical Sciences | 755 | 71 |
| 114 | University of Texas Health Science Center at Houston | 672 | 95 |
| 115 | Southern Illinois University | 656 | 78 |
| 116 | Madigan Healthcare System | 625 | 64 |
| 117 | John Peter Smith Hospital (Tarrant County Hospital District) | 625 | 44 |
| 118 | Naval Medical Center (San Diego) | 621 | 38 |
| 119 | Maimonides Medical Center | 610 | 29 |
| 120 | Orlando Health | 589 | 59 |
| 121 | Wright State University | 569 | 59 |
| 122 | Mayo Clinic College of Medicine (Arizona) | 549 | 63 |
| 123 | New York Medical College at Westchester Medical Center | 531 | 38 |
| 124 | University of Missouri at Kansas City | 523 | 43 |
| 125 | University of Nevada School of Medicine (Las Vegas) | 520 | 42 |
| 126 | Rutgers Robert Wood Johnson Medical School | 506 | 40 |
| 127 | Ochsner Clinic Foundation | 504 | 41 |
| 128 | University of Florida College of Medicine Jacksonville | 494 | 23 |
| 129 | Atlanta Medical Center | 462 | 39 |
| 130 | St Mary's Hospital and Medical Center | 377 | 24 |
| 131 | Akron General Medical Center/NEOMED | 368 | 28 |
| 132 | UPMC Medical Education (Hamot) | 334 | 26 |
| 133 | Summa Health System/NEOMED | 330 | 27 |
| 133 | Dwight David Eisenhower Army Medical Center | 330 | 26 |
| 135 | University of South Alabama | 299 | 24 |
| 136 | Detroit Medical Center/Wayne State University | 283 | 41 |
| 137 | University of Tennessee College of Medicine at Chattanooga | 241 | 32 |
| 138 | Howard University | 233 | 28 |
| 139 | Albany Medical Center | 230 | 43 |
| 140 | SUNY Health Science Center at Brooklyn | 217 | 43 |
| 141 | Albert Einstein Healthcare Network | 199 | 18 |
| 142 | Cooper Medical School of Rowan University/Cooper University Hospital | 181 | 24 |
| 143 | Wayne State University School of Medicine | 165 | 16 |
| 144 | Monmouth Medical Center | 127 | 9 |
| 145 | Palmetto Health/University of South Carolina School of Medicine | 105 | 9 |
| 146 | University of Kansas (Wichita) | 88 | 16 |
| 147 | University of Puerto Rico | 74 | 7 |
| 148 | St Luke's Hospital | 72 | 22 |
| 149 | Seton Hall University School of Health and Medical Sciences | 70 | 36 |
| 150 | Marshall University School of Medicine | 70 | 18 |
| 151 | Western Michigan University Homer Stryker MD School of Medicine | 67 | 15 |
| 152 | Los Angeles County-Harbor-UCLA Medical Center | 42 | 4 |
| 153 | Kingsbrook Jewish Medical Center | 35 | 8 |
| 154 | Hofstra North Shore-LIJ School of Medicine | 11 | 1 |
| 155 | University of California (San Francisco)/Fresno | 3 | 2 |
| 156 | University of Arizona College of Medicine-Phoenix | 1 | 1 |
| 157 | McLaren-Flint | 0 | 2 |
| 158 | Louisiana State University (Shreveport) | 0 | 0 |
